# Routine Use of Microbial Whole Genome Sequencing in Diagnostic and Public Health Microbiology

**DOI:** 10.1371/journal.ppat.1002824

**Published:** 2012-08-02

**Authors:** Claudio U. Köser, Matthew J. Ellington, Edward J. P. Cartwright, Stephen H. Gillespie, Nicholas M. Brown, Mark Farrington, Matthew T. G. Holden, Gordon Dougan, Stephen D. Bentley, Julian Parkhill, Sharon J. Peacock

**Affiliations:** 1 Department of Medicine, University of Cambridge, Addenbrooke's Hospital, Cambridge, United Kingdom; 2 Clinical Microbiology and Public Health Laboratory, Health Protection Agency Microbiology Services, Addenbrooke's Hospital, Cambridge, United Kingdom; 3 The Medical School University of St. Andrews, North Haugh, St. Andrews Fife, United Kingdom; 4 The Wellcome Trust Sanger Institute, Wellcome Trust Genome Campus, Hinxton, Cambridge, United Kingdom; 5 Department of Pathology, University of Cambridge, Cambridge, United Kingdom; The Fox Chase Cancer Center, United States of America

Whole genome sequencing (WGS) promises to be transformative for the practice of clinical microbiology, and the rapidly falling cost and turnaround time mean that this will become a viable technology in diagnostic and reference laboratories in the near future. The objective of this article is to consider at a very practical level where, in the context of a modern diagnostic microbiology laboratory, WGS might be cost-effective compared to current alternatives. We propose that molecular epidemiology performed for surveillance and outbreak investigation and genotypic antimicrobial susceptibility testing for microbes that are difficult to grow represent the most immediate areas for application of WGS, and discuss the technical and infrastructure requirements for this to be implemented.

## Introduction

Advances in whole genome sequencing (WGS) [1], [2] have resulted in a reduction in the full economic cost of sequencing a typical bacterial genome to as little as £40 (from extracted DNA to genome sequence). In addition, the speed of sequencing is increasing, with the prospect in the near future of a reduction in the time taken to sequence a microbial genome from several days or weeks to just hours. The combination of low cost and rapid turnaround time will mean that pathogen WGS can cross the divide between microbial research and the practice of diagnostic microbiology [3]–[6]. This holds the potential to transform our understanding of the evolution of pathogens and the global spread of antimicrobial resistance, a problem identified by the World Health Organization (WHO) as one of the three greatest threats to human health [7]. This step change could also represent the most significant advance in diagnostic microbiology and surveillance since the advent of *in vitro* culture.

The aim of this article is to discuss the potential utility and impact of pathogen WGS as a routine tool for diagnostic and public health microbiology, together with the technical and infrastructure requirements for this to be realised. The healthcare system in England is used as an example, but the findings are transferable to other sufficiently resourced countries. The differences between the various sequencing technologies [8], [9] and the lessons learned from the use of WGS as a retrospective tool for scientific research have been reviewed elsewhere [10]–[18] and will not be discussed here.

## Overview of the Current Diagnostic Paradigm in Diagnostic and Public Health Microbiology

**Figure 1:**
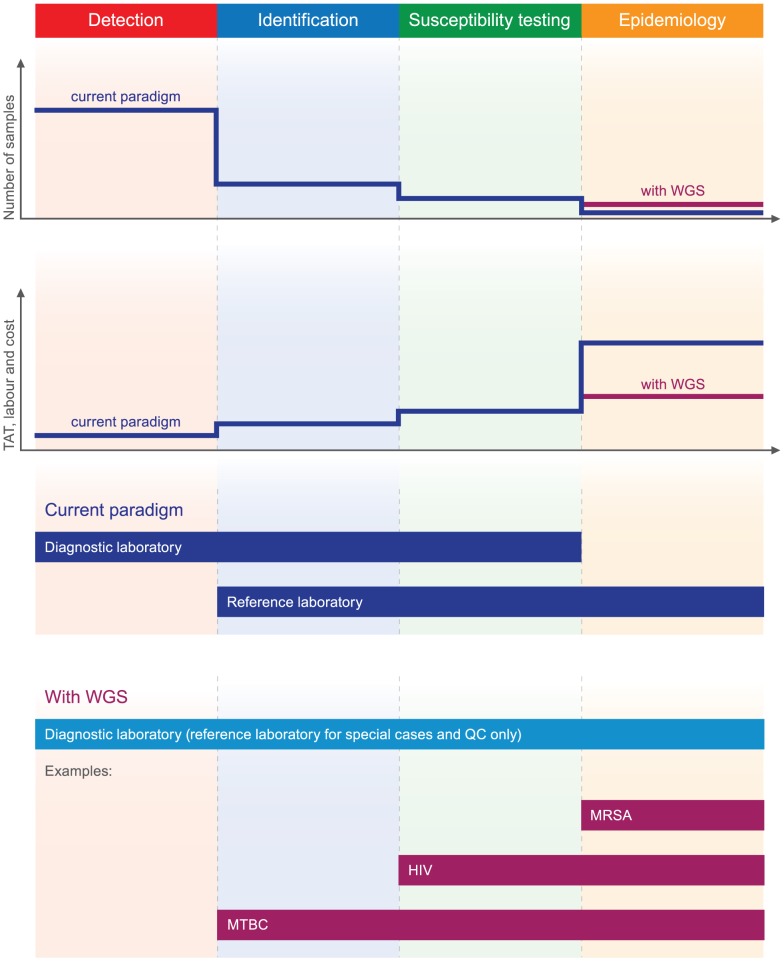
Potential impact of whole genome sequencing in diagnostic and public health microbiology. Clinical samples processed by diagnostic microbiology laboratories commonly pass through up to four stages, characterised by a stepwise decrease in the number of samples analysed at each successive stage and an inverse association with turn-around time (TAT), labour, and costs (shown in dark blue). Detection, identification, and susceptibility testing in virology are achieved using serological or molecular methods, whereas bacteriology generally relies on phenotypic methods. Epidemiological typing is only done for a handful of organisms using molecular and sometimes phenotypic methods. The most compelling immediate applications for WGS are molecular epidemiology for the purposes of surveillance and outbreak investigation (e.g. for MRSA) and drug susceptibility testing for organisms that are either slow growers or difficult to culture (e.g. MTBC and HIV). This is likely to lead to more samples being typed than is currently the case (depicted in purple), all while drastically reducing turnaround times, provided that WGS is performed in a regional or local laboratory rather than a reference centre. Assuming that this information enables cost-effective clinical interventions, the number of pathogens sequenced is likely to increase over time. Approaches to detection and identification of pathogens and the majority of susceptibility testing are likely to remain largely unchanged in the near future.

Deciding where to employ pathogen WGS in routine diagnostic microbiology requires consideration of the processes used in current laboratory practice. In very broad terms this is made up of four main stages, starting with detection (or not) of a pathogen in a sample. If a clinically relevant pathogen is detected, then this may be further tested for identification, drug susceptibility, and epidemiological typing. This simplified description best fits bacteria and fungi and is less accurate for viruses. Detecting the presence of a virus and species identification are often performed by the initial test (for example, a species-specific PCR), and susceptibility testing and typing are not performed for many of the viruses detected in the routine laboratory. But taken overall, each stage represents a reasonably circumscribed activity in routine laboratories, each of which is associated with a step-wise decrease in the number of samples analysed and an inverse association with turnaround times, labour, and costs. We have represented these features in a schematic illustration in Figure 1, the purpose of which is to provide a comparator (used later in the article) against which to describe the possible impact of WGS.

### Detection and Identification

Unlike diagnostic virology, which has already undergone a major shift from phenotypic to PCR-based genotypic tests, the detection of most bacteria and fungi still relies on culture-based methods developed for the most part over a century ago (exceptions include molecular assays such as those used to detect *Chlamydia*, methicillin-resistant *Staphylococcus aureus* (MRSA), and drug-resistant *Mycobacterium tuberculosis*, discussed below). What has changed recently is the degree of automation available for bacterial culture [19]. Agar plates can now be delivered, inoculated, incubated, and imaged by fully automated conveyor belt systems, reducing the amount of labour required at each of the multiple steps involved in sample processing. This development has been driven by the need to reduce costs while maintaining or increasing throughput, resulting in an on-going transition towards laboratory amalgamation and the expansion of centralised facilities, adoption of around the clock working practices, and a shift in the workforce skill mix towards a greater reliance on assistants and a reduction in the number of qualified laboratory technicians.

Organism culture is both cheap and appropriate for first-line microbiological screening processes. The turnaround time is around 1–2 days for many samples, although longer for those samples that require more prolonged incubation (such as blood cultures). For most specimens, this initial detection step is also the last as the majority of samples are reported as “negative” or “no significant growth.” For example, MRSA screening of 1,662 patients admitted to Addenbrooke's Hospital over a period of 1 week identified only 22 carriers (1.3%).

Once a significant bacterial or fungal isolate is detected, species identification is almost always performed. This may provide early prediction of antimicrobial susceptibility, clues as to the potential for disease progression, and can direct further investigations and clinical management. Identification is traditionally achieved using a range of approaches including manual kits (e.g. latex agglutination for *Staphylococcus aureus*). More recently, the emergence of MALDI-TOF has permitted accurate species identification within minutes directly from a single colony at only a fraction of the cost of traditional techniques and is likely to become the default front-line identification tool in clinical bacteriology [20], [21]. Moreover, there is a drive to apply this technology directly to samples from positive blood-culture bottles and, in some cases, even to primary samples [22].

A small number of pathogens require more extensive testing because of their pathogenic potential or infection control importance. This includes more detailed identification of certain organisms, or detection of specific virulence factors or toxins. For example, *Bordetella pertussis* infections are relatively rare in the United Kingdom but require rapid identification, confirmation, and characterisation. Much of this workload is handled by specialist reference laboratories, and usually adds a week to the turnaround time, although other tests can take considerably longer.

### Drug Susceptibility Testing

Drug susceptibility testing is currently undertaken for significant bacterial pathogens using standardised methods such as disk diffusion or automated systems. This process adds at least 1 further day to the turnaround time but can take significantly longer for some organisms, as discussed in the next section. In contrast, susceptibility is not routinely performed for the vast majority of viruses. HIV is the most prominent exception, which is performed using Sanger sequencing and typically takes between 1 and 2 weeks (J. Greatorex, personal communication). HIV genotyping will be discussed below.

### Epidemiological Typing

Microbial genotyping is also carried out to support infection control teams investigating putative hospital-associated outbreaks involving pathogens such as MRSA. In some cases, extended antimicrobial susceptibility patterns can provide timely evidence of the introduction and transmission of a new strain if it has a different susceptibility pattern to those seen in the preceding weeks or months [23]. In most cases, however, outbreak investigations must be supported by reference laboratory genotyping, the protocol for which is limited by three factors. First, isolate collection and batching introduces a delay; second, the turnaround time of at least 1 week for the test itself introduces a further delay (and together, these factors mean that genotyping information does not directly inform the management of the patient from whom the isolate was obtained, or of their contacts); and third, the current tools such as *spa*-typing and pulsed-field gel electrophoresis have a limited resolution, particularly to differentiate strains belonging to the same bacterial clone (such as EMRSA-15, currently the dominant hospital-associated MRSA clone in the United Kingdom [24]).

## Uses for Routine Pathogen WGS

### Epidemiological Typing

An obvious application for WGS is epidemiological typing to detect laboratory cross-contamination, to define transmission pathways of pathogens, and to support outbreak investigations [25]. Current bacterial genotyping techniques have a limited resolution because they only interrogate small regions of the microbial genome [14], [26], [27], whereas sequencing of the entire genome provides the ultimate resolution for epidemiological studies, as demonstrated by several recent studies including outbreaks of cholera in Haiti and *E. coli* O104:H4 in Germany [27]–[50]. For organisms whose rate of genomic change is sufficiently high, the resolution obtained may make it possible to reconstruct transmission pathways between healthcare centres, hospital wards, or even patients on the same ward [23], [51]–[57]. This would provide a mechanism for monitoring outbreaks in real-time and highlight daily opportunities for infection control [23], [58]. However, well-designed studies are required for each pathogen to determine whether routine use of WGS would be cost-effective.

### Drug Susceptibility Testing

The role of pathogen WGS in antimicrobial susceptibility testing is more limited. This is because the sensitivity and robustness of phenotypic susceptibility testing will be difficult to match with genotypic tests, in part due to incomplete data linking genotype to phenotype. Moreover, phenotypic testing is inexpensive. In the event that WGS was adopted for epidemiological purposes and was significantly faster than the 18–24 hours taken for standard disc susceptibility testing, then it could complement phenotypic testing, which would still be necessary to detect resistance encoded by novel mechanisms. For example, it could be used to rule in resistance for certain antibiotics where known drug-resistance mutations or genes are found before phenotypic results become available [23]. Where discrepancies between the antimicrobial susceptibility genotype and phenotype occur, phenotypic results could be repeated as part of a process to establish the basis for this. WGS as a sole diagnostic method to detect resistance is only viable where complete or near-complete congruence exists between phenotype and genotype, and where phenotypic testing is prohibitively slow. There are two good examples where this is the case.

The first relates to slow-growing bacteria, amongst which the *M. tuberculosis* complex (MTBC), the causative agent of tuberculosis, is the most prominent example. The slow growth rate of MTBC means that the turnaround time of full phenotypic susceptibility testing is measured in weeks, but its genomic homogeneity and the fact that resistance can only arise through point mutations or small insertions/deletions makes it an ideal target for genotypic testing [59]. Probe-based hybridisation tests have been used for a number of years, but their utility has been limited because they interrogate only a small number of loci responsible for drug resistance and are relatively labour-intensive [60]. More recently, the WHO has endorsed another genotypic test, namely the Cepheid Xpert MTB/RIF assay, which can simultaneously distinguish MTBC from other acid-fast bacteria and detect rifampicin resistance. This is achieved in an automated fashion directly from sputum within 2 hours [61], [62] and provides rapid information to the treating clinician and infection control team. This test could be complemented by WGS of the cultured organism (i.e. from a positive MGIT culture tube), which could, over time, replace the remaining diagnostic functions including the precise species identification, susceptibility testing for the remaining antibiotics, and epidemiological typing, which are currently achieved using a myriad of techniques at reference laboratories (Figure S1) [58], [63].

The second example relates to viral genotyping [64], [65], most importantly for HIV. Tests that determine viral tropism (which receptor HIV uses to enter cells) are required before the use of a cell entry inhibitor (e.g. Maraviroc), since these drugs are only active where HIV uses a specific receptor [66]. Current genotypic tests that rely on traditional Sanger sequencing technology are faster and cheaper than phenotypic tests, but are also less sensitive where a heterogeneous (genetically mixed) viral population exists. But given the ability to sequence every base position hundreds or even thousands of times, and thus detect minor variants within the population, current and future generations of WGS technologies hold the potential to match the performance of phenotypic assays [67]–[69]. Furthermore, drug resistance to other anti-retrovirals that evolves throughout treatment might be detected earlier by WGS, allowing the physician to alter the treatment accordingly [8], [70], [71]. In fact, this transition is already underway. Siemens has announced its intention to make the Trugene HIV-1 Genotyping Assay, which analyses the protease and reverse transcriptase coding regions of HIV, compatible with the Illumina MiSeq [72], [73].

### Detection and Identification

The potential overall impact on the diagnostic laboratory is summarised in Figure 1. As currently configured, it is unlikely that that WGS will be suitable for the routine primary detection of a pathogen and is unlikely to be applied directly to a clinical sample, as it would struggle to detect a pathogen with a low copy number or that is mixed with the normal microbiota [74], without an enrichment step for pathogen DNA as envisioned by Pathogenica [75]. Moreover, current detection tests are generally cheap and fast enough to satisfy the clinical need. Nevertheless, metagenomic research is beginning to suggest possible scenarios in which the relatively high cost of WGS for detection may be justified [12], [76]. For example, it has been shown that changes in the intestinal microbiota precede bloodstream invasion by vancomycin-resistant *Enterococcus faecium*, and monitoring of at-risk patients might provide novel opportunities for treatment intervention [77], [78]. Another potential early application of WGS is the accurate detection of non-culturable or difficult-to-culture organisms, including fastidious bacteria and anaerobes [79]. WGS could also be used in cases where standard diagnostic tests consistently fail to identify the causative pathogen, either because it is completely novel or because it is a variant of a known pathogen that leads to false negative results [16], [80]–[82]. In these cases, cheaper PCR-based tests could then be rapidly developed and deployed, as occurred during a recent outbreak of *Klebsiella pneumoniae*, with the carbapenemase enzyme OXA-48, which occurred in the Netherlands [12], [43], [81], [83].

Lastly, WGS could replace current PCR-based tests for toxins, as recently demonstrated for MRSA [23], if the bacterium in question was already sequenced for infection control purposes. Similarly, it could be used when MALDI-TOF does not offer the necessary resolution. Most prominently, MALDI-TOF cannot distinguish the various serogroups of *Salmonella* [21], yet the identification of enteric fever is crucial for treatment. WGS for such a narrow purpose alone may not be cost-effective, but could become a viable option if the genome data were also used for epidemiological purposes.

## Technical Requirements for Routine Pathogen WGS

The current generation of sequencers are designed to sequence human genomes. For WGS to be cost-effective for much smaller bacterial genomes, several hundred samples have to be batched, individually tagged (allowing bioinformatic designation of sequence back to an individual isolate), and then pooled at equimolar amounts. In contrast, clinical practice demands that the batch sizes are reduced to a minimum to suit the throughput of a diagnostic laboratory. To streamline this process further, sample preparation for WGS must be simplified, which would reduce the turnaround time to a few hours and the need for high-grade technical staff. In addition, the read-lengths generated must be sufficiently long to detect linked resistance mutations [8].

Lastly, WGS must become sensitive enough to sequence DNA from a single colony without the need for sub-culturing or a DNA pre-amplification step, saving a day of processing time. In this scenario, a single colony could be picked for phenotypic antimicrobial susceptibility testing, and the remainder used to extract DNA followed by overnight sequencing. As a result, both phenotypic susceptibility and whole-genome epidemiological typing results could become available in as little as 48 hours from the time of sample arrival. Existing and near-market platforms such as 454, Pacific Biosciences, Ion Torrent, and Illumina MiSeq promise to meet some of these targets but further evaluation is needed to assess their utility for routine clinical practice as opposed to their roles as tools for research [84]. With regard to the ease of sample preparation Oxford Nanopore promises to be very interesting for pathogen WGS [85].

If the above benchmarks are met, this will deliver tangible clinical benefits, all while simplifying the workflows of diagnostic laboratories. Given the universality of pathogen WGS, a wide diagnostic repertoire can be offered by a single machine that will replace multiple different methodologies, thereby allowing economies of scale. In this context, it is noteworthy that WGS analyses a sample in a single experiment as opposed to requiring multiple subtests. For example, the current HIV genotyping assay requires seven separate Sanger sequencing reactions to cover approximately 1.3 kb of sequence (J. Greatorex, personal communication). If one of them fails, the specific reaction might have to be repeated, thereby complicating the normal workflows that are key to ensuring the efficient use of laboratory resources. WGS would analyse the aforementioned stretch of the HIV genome in a single step, removing the potential for this complication. The same consideration applies to many of the current typing techniques such as MIRU-VNTR typing for MTBC (Figure S1).

An important question is where microbial WGS for diagnostic and public health purposes will be performed. Regional sequencing hubs have been developed in several centres across the United Kingdom and contain the technical expertise required for WGS, but alternative models include centralisation (a specialist reference laboratory) and decentralisation with a sequencing platform in most diagnostic laboratories across the country. This situation is likely to be fluid and change as the technology and interpretation tools develop and mature. Wherever it is performed, tight quality control of the entire process will be essential, as others have discussed in more detail [46], [86], [87].

## Pathogen WGS Data Interpretation and Use

**Figure 2:**
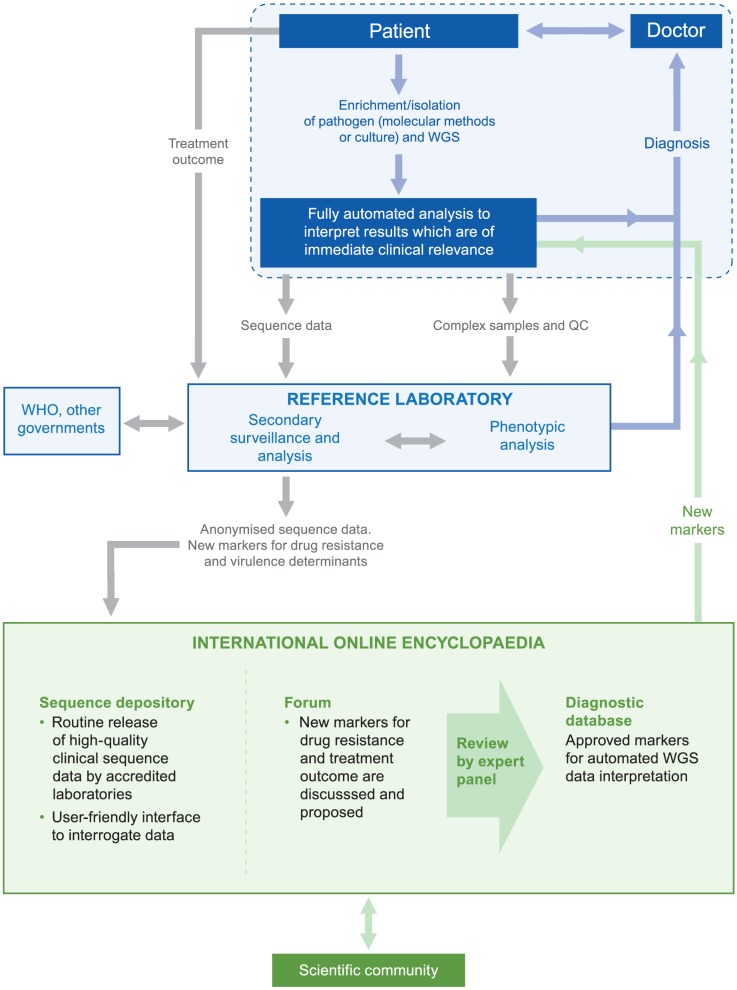
Overview of pathogen WGS data interpretation and use. After an enrichment step for pathogen DNA via culture or direct molecular enrichment/amplification (e.g. by RT-PCR for HIV) from the clinical sample, WGS could be performed in regional sequencing hubs. Fully automated analysis software would extract information of immediate clinical importance (i.e. an epidemiological analysis, susceptibility testing, and/or the precise identity of the pathogen). The results would be presented such that no knowledge of genomics would be required. Only in cases of new and emerging types of concern, where the genotype-phenotype relationship was unclear, or for quality control (QC) purposes would samples be sent for analysis by reference laboratories. Reference laboratories could routinely make anonymised sequence data available in the first part of an international online encyclopaedia to spur basic as well as translational research via a user-friendly interface to analyse whole genomes. Moreover, they could perform routine *in silico* surveillance of all genomes in their respective countries to relate treatment outcomes to particular genotypes or mutations. Clinically relevant surveillance results such as the discovery of novel pathogens or new markers for drug-resistance could be shared with appropriate international bodies and ultimately deposited in the encyclopaedia [101]. A forum could provide an opportunity for the scientific community to contribute and discuss findings with clinical implications. Novel markers would require review by an expert panel and, if approved, be incorporated into the read-only diagnostic database containing a catalogue of known resistance markers, which would then be used clinically by the automated WGS interpretation software.

With commercial companies in strong competition to develop and market accurate and affordable sequencing technologies, the major barrier to their practical implementation will shift from the issue of hardware to the software required to analyse the sequence data generated [25], [52], [86], [88]–[92]. Whereas traditional diagnostic tests yield either binary outputs or at most a handful of data points, WGS generates huge amounts of data [3]. Even with current Sanger sequencing which analyses relatively short stretches of DNA, human error rather than sequencing error is often the source of mistakes in genotyping [86]. Crowd-sourcing (as occurred for *E. coli* O104:H4 outbreak [43]) to analyse routinely sequenced bacteria is not fit for clinical practice given that the time taken for the analysis would be significantly longer than WGS itself, thereby reducing the time during which clinical interventions to contain outbreaks are possible. Similarly, having dedicated bioinformaticians in every diagnostic laboratory is not realistic. Instead, analysis software for WGS is required to extract clinically relevant information in a fully automated and reliable fashion without human intervention (Figure 2) [18].

Ideally, this software should have two key features. First, it should be platform independent, analysing sequence data directly without being tied to proprietary data formats. This would maintain flexibility and reduce to a minimum the lag time between the evaluation of new sequencers and their clinical use. Second, it should be organism independent, and be able to perform different tasks ranging from epidemiological tracking to susceptibility testing of any organism. In practice, first-generation interpretation technology is most likely to be developed for a small number of key organisms (e.g. HIV, MRSA, and MTBC) to which other organisms will be added over time.

The utility of interpretation software will depend on a continuously updated database that would not only encompass pathogen genomes to detect transmission (or lack thereof) but would also contain a catalogue of point mutations or genes that account for drug resistance. Samples that harbour previously unknown mutations in genes that confer drug resistance or other clinically relevant bacterial factors could be flagged for phenotypic follow-up by reference laboratories. Such information should be shared internationally [93] (as is already the case for the breakpoints for bacterial susceptibility testing [94] or HIV drug resistance mutations [95]), along with the routinely collected clinical and epidemiology data of WGS samples. Potential obstacles to their release include privacy concerns [96], questions of authorship [97], ownership, and intellectual property [98]. These will need to be tackled to maximise the possible healthcare benefits for individual patient care and regional, national, and international public health, as well as providing information to the scientific community to propel the rate of progress in basic and translational research [43], [96], [99].

One solution is the creation of an international, online encyclopaedia divided into three parts (Figure 2). The first part is a depository into which accredited diagnostic laboratories routinely deposit high-quality WGS data (i.e. draft genomes sequenced to a certain minimum depth and sequence quality [100]) from across the world [49], [58], [83], [87], [101], as pioneered by current MLST websites [102], the NCBI Influenza Virus Sequence Database [103], and the UK HIV Drug Resistance Database in which approximately 90%–95% (>51,000 tests) of all HIV genotypic drug resistance test data in the United Kingdom are represented [104]. This would include an open access user-friendly interface to reconstruct phylogenetic trees of whole genomes or single genes of interest. This could be coupled with a layered approach in which specific users have access to additional information. For example, national public health organisations could link clinical and genomic data for patient groups to provide the opportunity for more detailed analyses, while international organisations such as the European Centre for Disease Control or the WHO could have access to genomic information and limited anonymised clinical data from multiple countries to monitor cross-border spread of microbial pathogens and coordinate international interventions as appropriate [87], [101]. Online storage of only anonymised data would protect patient confidentiality. Nevertheless, making such information publicly available in a routine fashion is controversial, particularly since this might deter patients from seeking testing for some diseases. Given the imminent transition of WGS into the clinic we would encourage a timely debate of this issue by patients and their advocates, scientists, clinicians, ethicists, and policy makers [105].

The second part of the encyclopaedia could provide a forum to discuss new hypotheses (as piloted by *PLoS Currents: Influenza*) and, ultimately, to propose new markers for *in vitro* drug resistance, to link the genotype directly to disease outcome, or to identify novel pathogens [106], [107]. When warranted, new markers could be reviewed by an expert panel and added to the third part of the encyclopaedia, a read-only diagnostic database that contains a catalogue of all of the known mutations or genes of clinical importance. This process could be modelled after the European Committee on Antimicrobial Susceptibility Testing (EUCAST), which has used standardised methodologies to successfully harmonise and regularly review breakpoints for phenotypic susceptibility testing across Europe [94], and the Stanford HIV Drug Resistance Database [95]. The proposed encyclopaedia represents an infrastructure project that would require sustained funding [87], [91], [108].

## Implications for Reference Laboratories

The implementation of WGS as a routine clinical tool as well as the collation and expert oversight of these data by a public health organisation would represent a very powerful sentinel surveillance system. In theory, this could be funded by resources released by a reduced need for the phenotypic and genotypic tests currently performed by reference laboratories, although such a transition would be complex. Future central activities could include monitoring of the emergence, mechanisms, and global transmission of antimicrobial resistance, and the relationship between microbial genetic markers and patient outcome. Access to multiple genome sequences would also provide the opportunity to assess the conservation of potential targets for drugs or vaccines [14], [35], [109]–[111].

The following example gives a clear demonstration of the potential of this new paradigm: a recent study has found that strains of MRSA that cannot be detected by the current PCR assay targeting *mecA* have been circulating at a low frequency in the United Kingdom and Denmark for at least 36 years [81]. In a scenario where WGS was in widespread use, the genome sequence data together with periodic comparisons of genotype with phenotype would have identified such isolates. This could have triggered a national prevalence survey, experimental characterisation, and timely improvements in assays to close the gap in detection capability. Reference laboratories could call upon the isolating laboratories to supply samples of interest to perform targeted research to gain vital insights into new and emerging pathogens before they cause major outbreaks rather than merely reacting to these events. To enable this process, it will be necessary for diagnostic laboratories to store their bacterial isolates, at least temporarily, which is not always routine at present.

## Conclusion

WGS represents the opportunity for a step-change in diagnostic microbiological practice that in the long term could be associated with little or no increase in overall cost. Although the sequencing technologies will change over time, whole genome microbial epidemiology represents the ultimate source of information and will not be superseded. However, WGS will only replace some of the current diagnostic tests and will initially be targeted to specific samples or pathogens based on provision of comparable information to that currently obtainable but with a reduced turnaround time and/or cost, or the generation of information that is not currently available. This will require the development of fully automated sequence interpretation software, the provision of clinically relevant information in a format that can be understood and acted upon by healthcare workers with no specialist knowledge of genome sequencing, and the creation of systems for gathering and interpreting genome data at a population and public health level. In an age in which the further spread of drug resistance and the emergence of new pathogens are predicted [112], we can equip ourselves with the tools to detect, monitor, and control these threats to human health in real time.

## Supporting Information

Figure S1 Current diagnostic paradigm for MTBC compared with the use of WGS. According to the current diagnostic paradigm for *Mycobacterium tuberculosis* complex (MTBC), clinical samples (usually sputum) are first analysed using smear microscopy to detect high numbers of acid-fast bacilli. In parallel, cultures are inoculated (usually in liquid MGIT cultures), which yield positive results within 1 to 6 weeks. Positive cultures are then re-examined using smear microscopy to rule out contaminants or false-positive results and sent to a reference laboratory for speciation using molecular techniques such as DNA-hybridisation. These assays can also be used to detect drug resistance but have only been able to partly replace phenotypic tests because they target a limited number of resistance loci [60]. Similarly, the small number of DNA probes in commercial assays used to identify the precise member of MTBC results in the misclassification of some species or sub-species. Most prominently, only some but not all strains of *M. canettii*, which are intrinsically resistant against pyrazinamide, and potentially the novel agent PA-824 can be identified [113]–[115]. Therefore, phenotypic testing is still required. Some epidemiological typing techniques can be performed directly from the clinical sample, but in practice, they are generally performed at reference laboratories (figure adapted from Future Microbiology 2008; 3: 405–13 [60] based on [116], [117] with permission of the authors and Future Medicine Ltd.). In a future WGS paradigm, all functions could be performed in regional laboratories. First, the Cepheid Xpert TB/RIF test in combination with smear microscopy could be used to rapidly distinguish MTBC from other acid-fast bacteria and to detect rifampicin resistance. Provided that the resistance mechanisms for the various anti-tubercular drugs are elucidated more fully than is currently the case [118], WGS directly from the initial MGIT liquid culture could not only identify the precise sub-species but also detect resistance to all remaining drugs, and allow for epidemiological studies at the ultimate resolution [28], [37], [63].
(TIF)
