# Supplementary figures and images for: Routine Use of Microbial Whole Genome Sequencing in Diagnostic and Public Health Microbiology
Source: PLoS Pathog. 2012 Aug 2;8(8):e1002824. doi: 10.1371/journal.ppat.1002824 (PMC3410874; doi:10.1371/journal.ppat.1002824)

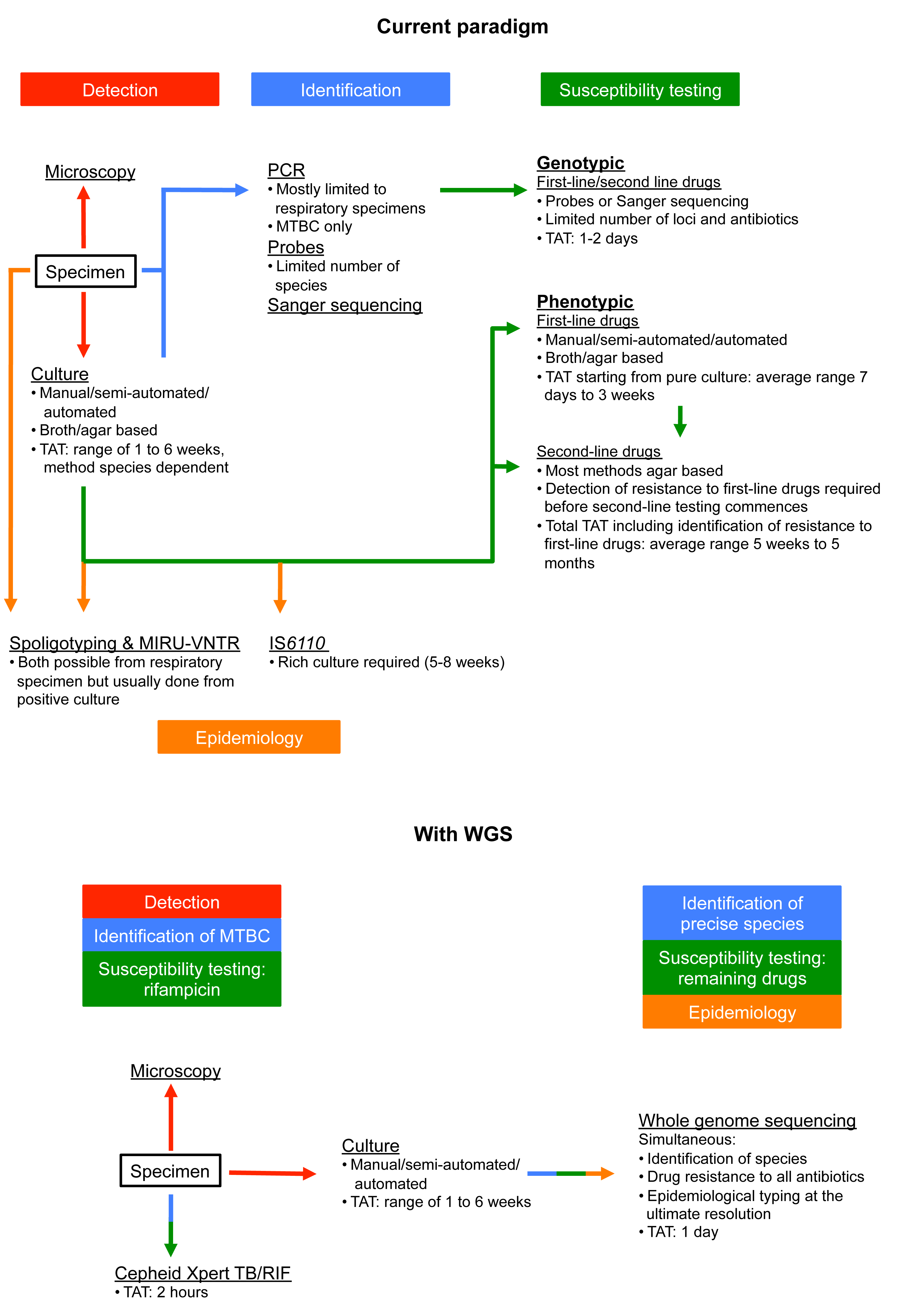

Supplement: Figure S1 — Current diagnostic paradigm for MTBC compared with the use of WGS. According to the current diagnostic paradigm for Mycobacterium tuberculosis complex (MTBC), clinical samples (usually sputum) are first analysed using smear microscopy to detect high numbers of acid-fast bacilli. In parallel, cultures are inoculated (usually in liquid MGIT cultures), which yield positive results within 1 to 6 weeks. Positive cultures are then re-examined using smear microscopy to rule out contaminants or false-positive results and sent to a reference laboratory for speciation using molecular techniques such as DNA-hybridisation. These assays can also be used to detect drug resistance but have only been able to partly replace phenotypic tests because they target a limited number of resistance loci [60]. Similarly, the small number of DNA probes in commercial assays used to identify the precise member of MTBC results in the misclassification of some species or sub-species. Most prominently, only some but not all strains of M. canettii, which are intrinsically resistant against pyrazinamide, and potentially the novel agent PA-824 can be identified [113]–[115]. Therefore, phenotypic testing is still required. Some epidemiological typing techniques can be performed directly from the clinical sample, but in practice, they are generally performed at reference laboratories (figure adapted from Future Microbiology 2008; 3: 405–13 [60] based on [116], [117] with permission of the authors and Future Medicine Ltd.). In a future WGS paradigm, all functions could be performed in regional laboratories. First, the Cepheid Xpert TB/RIF test in combination with smear microscopy could be used to rapidly distinguish MTBC from other acid-fast bacteria and to detect rifampicin resistance. Provided that the resistance mechanisms for the various anti-tubercular drugs are elucidated more fully than is currently the case [118], WGS directly from the initial MGIT liquid culture could not only identify the prec [file ppat.1002824.s001.tif]
